# Artificial Intelligence Model Chatgpt-4: Entrepreneur Candidate and Entrepreneurship Example

**DOI:** 10.12688/f1000research.144671.1

**Published:** 2024-04-22

**Authors:** Muhammet SAYGIN, Mustafa BEKMEZCİ, Evren DİNÇER

**Affiliations:** 1 Mersin University, Mersin, 33940, Turkey; 2 National Defence University, Ankara, 06100, Turkey; 3 Aksaray Municipality, Aksaray, 68100, Turkey

**Keywords:** ChatGPT-4, Entrepreneurship, Entrepreneurship Handbook, Artificial Intelligence

## Abstract

**Background:**

Although artificial intelligence technologies are still in their infancy, it is seen that they can bring together both hope and anxiety for the future. In the research, it is focused on examining the ChatGPT-4 version, which is one of the most well-known artificial intelligence applications and claimed to have self-learning feature, within the scope of business establishment processes.

**Methods:**

In this direction, the assessment questions in the Entrepreneurship Handbook, published as open access by the Small and Medium Enterprises Development Organization of Turkey, which focuses on guiding the entrepreneurial processes in Turkey and creating the perception of entrepreneurship, were combined with the artificial intelligence model ChatGPT-4 and analysed within three stages. The way of solving the questions of artificial intelligence modelling and the answers it provides have the opportunity to be compared with the entrepreneurship literature.

**Results:**

It has been seen that the artificial intelligence modelling ChatGPT-4, being an outstanding entrepreneurship example itself, has succeeded in answering the questions posed in the context of 16 modules in the entrepreneurship handbook in an original way by analysing deeply.

**Conclusion:**

It has also been concluded that it is quite creative in developing new alternatives to the correct answers specified in the entrepreneurship handbook. The original aspect of the research is that it is one of the pioneers of the study on artificial intelligence and entrepreneurship in literature.

## Introduction

“We call ourselves Homo Sapiens - man the wise - because our intelligence is so important to us.” ( Russell & Norvig, 2010). Looking at history, it is possible to see that various adversities that threaten the existence of human beings have been overcome with a new and innovative development and progress effort. All renewal efforts that were carried out after crisis periods such as wars, pandemics, and similar crises that affected all humanity actually formed today's scientific and technological infrastructure. Scientific and technological developments that accelerated after the Covid-19 pandemic have made change mandatory and opened the way for paradigm shifts in many sectors. In this process, organizations have questioned their crisis preparedness levels and started to search for new and innovative methods. Educational processes, in which activities independent of the place gain importance, have also been directly affected. In addition, the management styles and business establishment processes of enterprises have started to change in a similar way, with artificial intelligence technologies replacing the systems that allow remote working and learning today.

Entrepreneurship is the process of developing new concepts, goods, or services that lead to commercial prospects. Entrepreneurs take chances, put fresh ideas into practice, launch new firms, and work to expand them. In this process, business owners frequently need to locate funding, plan their ventures, develop marketing plans, and oversee operational tasks. There are many benefits and difficulties in the dynamic world of entrepreneurship.

When implementing their business ideas, entrepreneurs assume some risk. Financial, operational, and competitive hazards abound when beginning and expanding a new firm. In addition, there are unknowns in the realm of entrepreneurship. Entrepreneurs may need to adjust swiftly to changes in elements including market conditions, client preferences, and competitive environment. While starting and expanding their firms, entrepreneurs must also adjust to the fast-paced working environment. Over the past decade, there has been a notable surge in entrepreneurship research, encompassing diverse themes and issues. Artificial intelligence research is having both positive and negative effects on sustainable development ( Gupta et al., 2023). There is a glaring absence of systematization in academic literature regarding this correlation, despite the growing consensus regarding the disruptive potential of artificial intelligence (AI) and its favourable influence on entrepreneurship ( Giuggioli & Pellegrini, 2022). As Zen et al. (2023) emphasize, entrepreneurship education is crucial both in theory and practice.

It would be appropriate to explain what entrepreneurship means. Shane & Venkataraman (2000) define the entrepreneurship as the discovery and utilization of commercial opportunities within the individual-opportunity nexus while van Praag & Versloot (2007) emphasize the innovation, economic and societal growth of nations, and the creation of jobs all depend on entrepreneurship. As Naudé (2008) questions entrepreneurship’s role in economic growth, the possible response might be given as entrepreneurs help convert low-income, primary-sector communities into high-income service and technology-based society. Furthermore, it should elucidate the role of entrepreneurs in fostering both innovative growth and addressing stagnant development. The development of the private sector, entrepreneurship, and small and medium-sized businesses (SMEs) are at the centre of any country in transition's political and economic reform. SMEs are crucial to innovation and the high-tech industry, and many of them grew into huge corporations thanks to their adaptability and creativity ( Tan & Yıldıran, 2018).

Moreover, it is useful to express how important it is in the entrepreneurship of public institutions. In industrialized nations, the public sector is not solely responsible for job creation. Although recent studies indicate that even the largest and oldest private enterprises are unable to offer new chances to battle the high rate of unemployment, the private sector appears to play a significant role in finding solutions to the problem of unemployment. One such collaboration is known in Turkey as KOSGEB (Small and Medium Enterprises Development Organization). Through its Entrepreneur Support Program, KOSGEB, a state-owned organization, offers tremendous chances to aspiring entrepreneurs who want to launch a new firm ( Akpomi, 2018).

It is widely acknowledged that supporting women's entrepreneurship is essential for ensuring both economic and social progress. However, a lot of societal and financial obstacles prevent women from starting their own businesses. Despite these challenges, there are numerous governmental and non-governmental organizations as well as international organizations that assist and support women. One of these agencies and organizations, KOSGEB encourages female business owners ( Durukan, 2021). SMEs, which make up the majority of the Turkish economy and serve as its foundation, appear to be structures that are easily adaptable to change and competition in the modern world. their varying economic structures and sizes. However, both industrialized and emerging nations must recognize the potential they provide for nations, particularly in terms of employment and production. One of the main objectives of governments and state institutions is to improve the competitiveness and adaptability of SMEs, which are crucial for our nation and the rest of the world. SMEs are supported by a variety of aids provided by KOSGEB which was actually created to boost the share of SMEs in serving the requirements of people and businesses ( Kandemir et al., 2017).

The support provided by KOSGEB is crucial to the establishment, development, competition, and innovation processes of entrepreneurs and SMEs. These assistance and educational programs aid companies in overcoming financial challenges, gaining a competitive edge, and achieving sustainable growth. In the study, the Entrepreneurship Handbook, which is very important for business owners and is provided by KOSGEB as open access to all of its stakeholders, was looked at, and the evaluation questions were assessed using an artificial intelligence model. Thus, the effectiveness of ChatGPT-4, an artificial intelligence application that exemplifies entrepreneurship, as an entrepreneur candidate has been established.

## Literature review

A field called artificial intelligence is intended to give computer systems human-like intelligence. Artificial intelligence is seen to be capable of carrying out tasks including pattern recognition, problem solving, learning, and decision-making based on data analysis. This technology is employed across a wide range of industries, including automotive, health, finance, and education. According to the formal definition, the term “artificial” refers to something that is made by humans by analogy with natural examples. The term “intelligence” refers to all the cognitive abilities of an individual, including thinking, reasoning, perception of objective facts, judgment, and drawing conclusions ( Turkish Language Association, 2023). Artificial intelligence (AI) is a concept that emerged in the 1960s and is considered one of the fundamental concepts of Industry 4.0 philosophy. AI encompasses a range of methods for solving complex problems arising from the intelligent behaviour of computers ( Skrop et al., 2018). AI involves the use of computer programs to perform problem-solving tasks that humans traditionally perform. One of the newest areas of science and engineering is artificial intelligence. Currently, AI covers a vast array of subfields, from the broad to the narrow ( Russell & Norvig, 2010).

Since the invention of artificial intelligence in 1956, there have been two opposing paradigms: symbolism and connectionism (or sub-symbolism). By the end of the 1980s, symbolism predominated AI research, but connectionism gained traction in the 1990s and is now steadily displacing symbolism ( Zhang et al., 2023). As Jiang et al. (2022) mention, over the past 65 years, scientists and engineers have been actively researching artificial intelligence. The straightforward argument is that machines made by humans are capable of becoming intelligent like humans and of performing tasks that require a lot of manual labour. Whether we are aware of it or not, artificial intelligence has a significant impact on how we live our lives, playing unique roles in a variety of public-facing sectors like business, healthcare, transportation, and education.

Industry 4.0, a paradigm incorporating contemporary technologies and advances, can now be regarded a reality. Artificial intelligence is the key driver of the industrial transformation because it enables intelligent machines to do functions like self-monitoring, interpretation, diagnosis, and analysis on their own ( Ahmed et al., 2022). Artificial intelligence is advancing rapidly at the moment and will soon become a man's unavoidable companion. The usage of software and technology nowadays significantly improves human capacities ( Aggarwal et al., 2022). In numerous situations, such as identifying street views, extracting roadways, and understanding medical images, artificial intelligence models have outperformed traditional data management. Many well-known theories and techniques emerged from the first generation of AI research in the 1980s, but due to computer constraints, the initial models were too laborious to train. AI has advanced scientific developments and discoveries in medicine, biology, and economics thanks to the recent rapid growth of hardware and software ( Sun et al., 2022).

Powerful language models like Chat Generative Pre-Training Transformer, Google's BARD, and Ernie, which have proven to be excellent at many diverse language tasks, have been made possible by recent advances in Natural Language Processing ( Rahaman et al., 2023). Baidoo-Anu & Owusu Ansah (2023) identify that ChatGPT made its first appearance in 2022 in the public domain and within a week had more than a million subscribers. The globe was taken aback by the advanced ability of the generative AI tool ChatGPT to complete remarkably complex jobs. Teachers have conflicting sentiments about ChatGPT's amazing capacity to carry out complicated tasks in the realm of education because this development in AI appears to revolutionize current educational praxis. The fourth version in OpenAI's numbered “GPT-n” series of Generative Pre-Trained Transformer foundation models, Generative Pre-Trained Transformer 4 (GPT-4) is a multimodal big language model which has become available on March 14, 2023 and published as the most advanced algorithm from OpenAI, generating safer and more helpful responses ( OpenAI, 2023).

The application of artificial intelligence application ChatGPT is being tried in many different fields and different findings are reached. It would be correct to say that the product has become a subject of interdisciplinary study apart from the effect it creates. For instance, the research in which AI has been treated as a medical doctor, it is emphasized that online medical consultations have grown in popularity recently so, an increasing number of people may inquire about their diseases, symptoms, and differential diagnoses using the lately popularized ChatGPT. During the research process, clinical case vignettes, including some uncommon case presentations, were used to test ChatGPT's diagnostic precision and found that ChatGPT 4 resolves every typical case in just two suggested diagnoses ( Mehnen et al., 2023).

Besides that, another research dealing with the engineering field analyses the possibility of ChatGPT’s passing the engineering exams. The authors state that with the introduction of OpenAI ChatGPT-4, the engineering community has recently witnessed the development of chatbot technology. It is investigated if these chatbots can pass the Fundamentals of Engineering Principles and Practice of Engineering exams, despite the fact that they have been claimed to perform well and even pass a variety of standardized tests, including the legal and medical exams. It seems clear that ChatGPT-4 in its current form has a chance of passing the Fundamentals of Engineering (FE) test ( Naser et al., 2023).

While Nune et al. (2023) discuss whether ChatGPT has the capacity to react to human instructions after collecting data, analysing the information, and using machine learning (ML) techniques, the research of Holterman & van Deemter (2023) in which argues the theory of mind which is the capacity to comprehend human thought and decision-making, clarify that ChatGPT-4 compared to the previous versions was demonstrated to arrive at the right answers more frequently than would be predicted simply on chance.

The use of language models in interdisciplinary studies can facilitate quick and effective access to information and support finding solutions to problems by bringing together expertise in different fields. Therefore, ChatGPT can be used to answer questions in a variety of disciplines, as the courses are trained based on a large dataset containing specialist knowledge from different fields and It can also be used interdisciplinary in written communication fields such as language models, text production and editing. It might be said that ChatGPT has already become the subject of interdisciplinary study; for example, Huang et al. (2023) try to benchmark ACR Radiation Oncology In-Training Exam, Törnberg (2023) deals with the Text analysis of Political Messages on Twitter, Teebagy et al. (2023) research the evaluation of Ophthalmology exam as well as Zhang & Gosline (2023) try to identify perception of the people by using ChatGPT. As Dowling & Lucey (2023) identified, for the researchers looking to develop new insights and ideas, ChatGPT might be useful tool.

## Methods

As mentioned by Fitria (2023), an artificial intelligence model, which can be regarded as a chatbot, is simple to use by logging in with an email address. Any question or statement can be entered into the available dialogue field, and ChatGPT will provide an instantaneous response. The study examined the Entrepreneurship Handbook, which is openly accessible on the official website of the Small and Medium Enterprises Development and Support Administration. This handbook comprises 16 chapters that cover various dimensions of entrepreneurship. The chapters were authored by scholars and sector representatives affiliated with universities in Turkey. At the conclusion of each chapter, under the heading “Let's test ourselves,” readers are presented with 10 questions accompanied by answer keys. The primary research approach employed was content analysis, a qualitative research method. The utilization of artificial intelligence to examine the questions individually served as a novel and distinct method. Consequently, the primary aim of the research was to assess the effectiveness of ChatGPT-4 in addressing entrepreneurship-related questions. The inclusion of all 160 questions within the research scope and their analysis through artificial intelligence application supports the conclusion that the census method is preferred.

The underlying assumption of the research is whether ChatGPT-4 can successfully answer entrepreneurship exam questions in Turkish without prior knowledge loading. Consequently, the hypotheses formulated within the scope of the research are as follows:

H1: ChatGPT-4 demonstrates success in the entrepreneurship exam.

H2: ChatGPT-4 can employ the self-learning method in the entrepreneurship exam.

H3: Language preference influences the success level of ChatGPT-4.

H4: ChatGPT-4 exhibits equal success in all subtopics of the entrepreneurship exam.

### Study design & data collection

In the process of data analysis, it was observed that all the questions were originally written in the native language (Turkish), and the analysis was conducted using the Turkish language. Subsequently, the questions and answers were translated into English language at the 2nd stage. Language experts proficient in English linguistics were involved in the translation process. The artificial intelligence application ChatGPT-4 was then utilized to translate the questions. The analysis procedure consisted of three stages. In the first stage, the accuracy of the answers provided by ChatGPT-4 was determined by comparing them to the correct answers. In the second stage, questions that were answered incorrectly were re-evaluated and reviewed. In the third stage, the questions that still received incorrect answers after the first two stages underwent a systematic process. Preliminary information regarding these questions was uploaded to the system, and the questions were re-examined. At the conclusion of the third stage, it was observed that ChatGPT-4 provided correct answers for all questions. The analysis process of the questions followed these steps.

Stage 1: Initial Analysis with Dataset

Stage 2: Re-analysis of Incorrect Answers

Stage 3: Final Analysis of Incorrect Answers with Content Assistance

In this process, specific commands are provided to the ChatGPT-4 model before each query. The pre-command used before answering the questions is as follows:

Stage 1: “Think like an aspiring entrepreneur and a scholar specializing in entrepreneurship. Analyse each question below thoroughly and provide the most appropriate answer along with its rationale.”

Initially, it was observed that ChatGPT-4 provided correct answers to 144 questions without any intervention. The fact that 144 correct answers were obtained in the initial application indicates the highly successful performance of the artificial intelligence model. However, to further evaluate its self-learning capability, a retest was conducted for the questions that were initially answered incorrectly, and the questions were redirected. It was observed that ChatGPT-4 tended to review the incorrect answers initially provided and offer alternative responses. Moving on to the second stage, the 16 questions that were previously answered incorrectly were compiled into a single file and reanalysed using the following pre-command. At this stage, questions were asked by using both Turkish and English languages.

Stage 2: “Think like an aspiring entrepreneur and a scholar specializing in entrepreneurship. Reanalyse each question, considering alternative answers, and provide the most appropriate answer along with its rationale.”

After identifying the content of the questions that were initially answered incorrectly in the Entrepreneurship Handbook and uploading them as paragraphs into the system, correct answers were obtained for all questions. In the third stage, the pre-command loaded into the system is as follows:

Stage 3: “Carefully read the paragraph. Rethink like an aspiring entrepreneur and a scholar specializing in entrepreneurship. Analyse each question below thoroughly and provide the most appropriate answer along with its rationale.”

Thus, the primary focus of the research is to determine the level of success of the ChatGPT-4 model in the entrepreneurship assessment and to examine language variations. Additionally, it aims to assess the significance of the self-learning ability in the

## Results

In this part of the study, the findings pertaining to the questions answered by ChatGPT-4 are presented. Throughout the research process, the questions from each chapters were initially compiled into separate files. Then, sets of 10 questions from each chapter were posed to the ChatGPT-4 model using the Turkish language. As it incorporates a self-learning method, no prior information was loaded when querying the model with questions related to the chapters. This means that the content of the questions was not uploaded into the system in any way, and direct questions were asked to obtain answers.

The dataset was analysed in three stages, and it was observed that correct answers were provided for all questions by the end of the third stage. The stages of the answering process are illustrated in the table below.

As indicated in Table 1, ChatGPT-4 initially demonstrated success in passing the Entrepreneurship exam. In the subsequent stages, the researcher attempted to identify all the correct answers based on the exam requirements. In the final round, it was observed that providing the relevant content of the questions increased the accuracy of responses. However, it would be more meaningful to analyse the questions posed to ChatGPT-4 in the first stage without introducing any “self-learning” conditions. Therefore, this chapter focuses on the interpretation of the data obtained in the initial stage of the research process. A total of 160 questions were asked, out of which 16 questions were answered incorrectly. The chapters of the Entrepreneurship Handbook are as follows:

**Table 1.** Stages of answering process.

Stages	Process	Correct answers
Stage 1	Initial Analysis with Dataset	144
Stage 2	Re-analysis of Incorrect Answers (Language Exchange)	150
Stage 3	Final Analysis of Incorrect Answers with Content Assistance	160

Chapter 1 - Basic Concepts of Entrepreneurship

Chapter 2 - Seeing Entrepreneurship Opportunities and Generating/Developing Ideas

Chapter 3 - Feasibility Analysis

Chapter 4 - Business Models, Customers, Value Propositions and Sources of Revenue

Chapter 5 - Economy, Industry, Competition and Customer Analysis

Chapter 6 - Legal Background

Chapter 7 - Ethical Foundations of the Enterprise

Chapter 8 - Marketing Principles and Management

Chapter 9 - Networking

Chapter 10 - Determination and Management of the Financial Structure of the Enterprise

Chapter 11 - Access to Financial Resources for Start-ups

Chapter 12 - Innovation Management

Chapter 13 - Intellectual Property Rights

Chapter 14 - Professional Management of Enterprise and Strategic Management in SMEs

Chapter 15 - Management of the Growth Process and Growth Strategies

Chapter 16 - Developing a Business Plan

**Figure 1.**
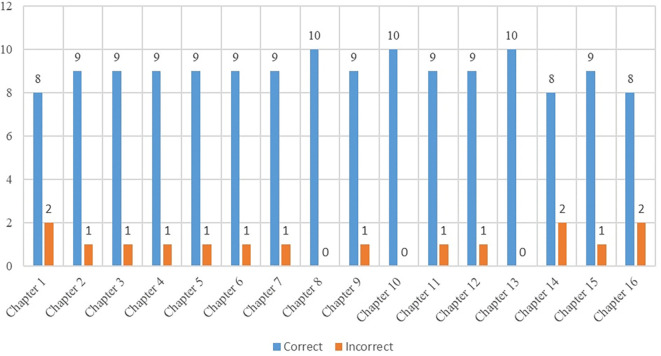
Correct answers of sections.

The correct answers of 144 questions were found by the artificial intelligence application ChatGPT-4 is shown in Figure 1.

As depicted in Figure 1, the language model exhibited a high level of success in answering the majority of the 160-question dataset across 16 chapters. Notably, in Chapter 8, focusing on Marketing Principles and Management, and Chapter 10, addressing the Determination and Management of the Financial Structure of the Enterprise, ChatGPT-4 accurately answered all questions in the first stage. This data, as shown in Figure 1, highlights the model's particularly strong performance in the areas of marketing and finance, where it achieved a perfect score from the outset. Nevertheless, it is crucial to examine some of the questions that ChatGPT-4 initially answered incorrectly. For instance, in Chapter 1 there were 10 multiple-choice questions. ChatGPT-4 correctly answered 8 out of the 10 questions in the first stage. When reviewing the two questions that were answered incorrectly, it is worth considering that the 8th question could possibly be deemed correct. According to Özdemir et al. (2007) businesses that employ 1-250 people or have a certain turnover or financial balance sheet are referred to as SMEs. Therefore, it is plausible to interpret the 8th question as correct, aligning with the mentioned definition of SMEs.

The 8th Question (Chapter 1): “How is the size of a business with 45 employees and a turnover of 20 million TL defined in our current laws?”

Correct Answer by Source: “Small Business”

Correct Answer by ChatGPT-4: “SME”

Upon examining the 8th question in the 1st chapter, according to the provided source, the correct answer is designated as “Small Business”. However, ChatGPT-4 identifies the correct answer as “SME”. When asked to explain the reasoning behind its answer, the artificial intelligence application indicates that a business with 45 employees and a turnover of 20 million TL falls under the category of a “medium-sized enterprise” based on both employees count and annual net sales criteria. It is worth noting that in Turkey, such businesses are also considered within the scope of SMEs. Considering the answer provided by both the source and the artificial intelligence, it can be inferred that a business with 45 employees and a turnover of 20 million TL can be classified as both a small business and an SME. Therefore, although there is a discrepancy in the correct answers, it can be argued that the artificial intelligence application has answered this question correctly by taking into account the broader definition of an SME.

10th Question (Chapter 1): “Which of the following is not a misconception about entrepreneurship?”

Correct Answer by Source: “No matter how valuable, the business idea alone has no value”

Correct Answer by ChatGPT-4: “If the business idea is very complex, success is guaranteed”.

Based on the data presented above, it appears that there is a discrepancy in determining the correct answer to the 10th question. Upon examining the source of the questions, it states that the statement suggesting that a valuable business idea alone will not have any value is not one of the well-known misconceptions about entrepreneurship. On the other hand, the artificial intelligence application, ChatGPT-4, attempted to identify the incorrect statements among the options by providing the following explanation: “There seems to be some confusion with the phrase (not one of the known mistakes), but let's try to identify the incorrect statements in the options'.”

Upon analysing the answers provided by ChatGPT-4, the following ideas can be inferred:

(a) A simple and understandable business idea has a better chance of success.
(b) Failure is a natural part of the entrepreneurial process, and individuals need to learn to cope with failure.
(c) Not every entrepreneur is inclined to take risks, and some prefer less risky business ideas.
(d) A good business idea can hold significant value and serve as the foundation for a successful enterprise.
(e) While money is undoubtedly an important factor in entrepreneurship, it is possible to establish a successful venture without it.

When examining the source material, specifically Chapter 1 - Basic Concepts of Entrepreneurship written by Özgül (2022) within the Entrepreneurship Handbook ( Erdoğan, 2022) it becomes apparent that there is a significant amount of outdated or incorrect information concerning entrepreneurship. Such material, often spread through word of mouth or the internet, can distort people's perceptions about entrepreneurship. It is crucial for both current business owners and aspiring entrepreneurs to be aware of these common misconceptions. The entrepreneurship myths outlined by Erdoğan (2022) are as follows:

Myth 1: The most important issue in the entrepreneurial process is the business idea.

Myth 2: Success is guaranteed if the business idea is very complex.

Myth 3: If no one has the product, success is guaranteed.

Myth 4: It is unnecessary to prepare a business plan if the business is to be established with equity.

Myth 5: Don't share your business idea with anyone.

Myth 6: The failed entrepreneur cannot be trusted

Myth 7: Entrepreneurs like to take risks

Myth 8: Entrepreneurship is for those with money

However, due to the ambiguity within the question itself, it has become difficult to determine the correct answer, resulting in ChatGPT-4 providing a response indicating confusion at the second stage: “There is some confusion with the expression, but let's try to identify the wrong statements in the options.” Therefore, ChatGPT-4 gave an incorrect answer for this question. It is important to acknowledge that the language used in the question itself lacks clarity in terms of its semantic purposes.

If we consider the questions that were answered incorrectly by the artificial intelligence application in general, it can be observed that the complexity of the business idea does not guarantee success. Likewise, it is incorrect to claim that an unsuccessful entrepreneur cannot be trusted, as failures often contribute to gaining valuable experience. Taking risks is a characteristic commonly associated with entrepreneurship. Additionally, the business idea is indeed a key element in the entrepreneurial process. It is incorrect to state that entrepreneurship is solely for those who have money since financial capital is just one component of the overall picture. Entrepreneurs also require intangible capital elements for success.

In summary, the inaccuracies in the incorrect answers provided by ChatGPT-4 highlight the importance of considering the nuances and complexities of language in order to arrive at accurate interpretations and responses. Samples of incorrect answers from other chapters of Entrepreneurship Handbook are as follows:

3rd Question (Chapter 2): “Which of the following is never asked during customer discovery?”

Correct Answer by Source: “We took out shampoo so that your dog does not smell bad, would you buy it?”

Correct Answer by ChatGPT-4: “What do you do if your dog smells bad?”

In the 2nd Chapter of the handbook, which focuses on entrepreneurial opportunities and generating ideas. it was found that only the 3rd question was answered incorrectly by the artificial intelligence application. Upon examining the relevant source, it was observed that different answers were provided by the application due to the use of expressions that are generally subjective and not backed by scientific citations within the chapter created by a private sector representative. Moreover, the question itself contains relative and subjective expressions. The question is presented as follows ( Ünsal, 2022):

“Imagine you produce a dog shampoo that gives canines a fantastic scent. You took the first prototype in your hands, but you also walked out on the street to get a better understanding of the customer. If you say, ‘Hello, can I ask you a few questions?’ to someone while they are walking their dog. Would you buy a dog shampoo that we created that makes your dog smell so good and stops them from smelling bad? To avoid offending you, they might answer ‘yes’ to this question, but you won't learn anything in this method.”

ChatGPT-4 initially failed to provide the correct answer for this question. However, when the question was revisited in the 2nd stage of the research, a different answer was generated. Therefore, although it is acknowledged that the question was initially answered incorrectly, it is believed that it is challenging to direct the response to a single correct answer due to the subjective nature of the question and the absence of a definitive solution in terms of common consensus.

4th Question (Chapter 3) “Customers don't buy a product, but buy the benefit that product provides.” What is the name given to the lack of this view, which forms the basis of value creation and the marketing view?

Correct Answer by Source: “Marketing Blindness”

Correct Answer by ChatGPT-4: “Product Blindness”

In the 3rd chapter of the handbook, which covers feasibility analysis and is written by Aygören (2022), ChatGPT-4 answered only 1 question incorrectly, similar to the previous chapter. However, it was observed that the artificial intelligence application provided comprehensive and insightful answers due to the details included in the responses, even for the question that was initially considered incorrect. At the 2nd stage of the research, ChatGPT-4 offered a reasonable explanation by stating that “product blindness is caused by the company's over-focus on its own products, while marketing blindness is caused by the company's inability to focus enough on the needs of its customers.”

Product blindness refers to a situation where a company becomes excessively focused on its own products during the product development phase. This can lead to the company having extensive knowledge about its products, including their functionality, features, and manufacturing process. However, customers may not possess this same level of understanding and may have different needs and preferences. As a result, the company may struggle to align its offerings with the actual needs of customers, leading to a disconnect.

On the other hand, marketing blindness occurs when a business fails to comprehend the needs and behaviours of its customers. Instead of prioritizing and addressing the real needs of customers, the company may overly emphasize the features of its products and overlook the genuine requirements of its target market. In essence, product blindness stems from the company's excessive focus on its own products, while marketing blindness arises from its failure to adequately prioritize and cater to the needs of its customers. Therefore, it can be concluded that ChatGPT-4's response, which highlighted the distinction between product blindness and marketing blindness, provides a reasonable and accurate understanding of these concepts.

6th Question (Chapter 4): “Which of the following is not a benefit of using business models?”

Correct Answer by Source: “Preparation of a long-term, comprehensive and detailed plan.”

Correct Answer by ChatGPT-4 “To be able to see what kind of alternatives the business idea can have”

4th Chapter focuses on business models, customers, value propositions and revenue streams ( Oran, 2022). When the questions within the scope of the chapter were examined, it was seen that 6th question was answered incorrectly by ChatGPT-4 by giving the reasons like: “One of the benefits of using business models is that alternatives to the business idea can be seen. Other options are among the benefits of using business models. Option (a) provides a clearer understanding of business ideas by putting them on paper. Option (b) ensures that important points are detailed without skipping. While option (c) helps devise a long-term plan (d) helps identify the assumptions necessary for the business idea to be successful.”

When considering the assessment questions in other chapters of the Entrepreneurship Handbook in general, the analysis was similarly completed with a maximum of two incorrect answer processes per chapter. In general, there were 16 incorrect answers in the first stage, while a decrease was observed in the number of incorrect answers in the second stage. The reason for this situation is thought to be the translation of the questions from Turkish to English, which may have eliminated the ambiguity. In the third stage, it was observed that the correct answers to the questions could be obtained by examining the chapters related to the incorrectly answered questions and transferring the sections with the answers to ChatGPT-4. When considering the research hypotheses, it was found that this chatbot application was successful in the entrepreneurship exam, as it succeeded in most of the questions that make up the dataset. Therefore, the hypothesis “H1: ChatGPT-4 demonstrates success in the entrepreneurship exam” was accepted.

As mentioned before, to determine whether ChatGPT-4 has a self-learning method, the dataset was examined in three stages. The processes of directing the questions, posing the questions by repetition and language change, and ultimately re-directing the questions with the help of the contents were followed. The increase in the number of correct answers obtained indicates the self-learning ability of the system. Therefore, the hypothesis “H2: ChatGPT-4 can employ the self-learning method in the entrepreneurship exam” was accepted.

Another assumption of the research is whether language preferences will affect the success of the artificial intelligence application in exam questions. The increase in the number of correct answers obtained by asking the 16 questions again in English that were answered incorrectly before can be considered as an indicator. However, it should be noted that much more data is needed to determine whether the questions translated from Turkish to English can be understood more clearly by ChatGPT-4 in terms of semantic and syntactic features. Therefore, it is worth mentioning that the clarification in the meaning units of the questions may be predicted by using different language structures together with the existing dataset. Hence, hypothesis 3 was neither fully accepted nor rejected based on the current dataset. The hypothesis that ChatGPT-4 shows equal success within the scope of entrepreneurship exam areas has been rejected. This is because there are sections in the first stage of the dataset analysis where all questions are answered correctly by ChatGPT-4. Therefore, all of the questions in Chapter 8 - Marketing Principles and Management and Chapter 10 - Determination and Management of the Financial Structure of the Enterprise have been answered correctly. With this finding, although it is possible to say that ChatGPT-4 is more successful in solving questions on marketing and finance, it is thought that whether each question is semantically understandable, regardless of the subject of the questions, is a phenomenon that should be examined by linguists.

## Discussion

In conclusion, artificial intelligence technologies hold both promise and anxiety for the future. ChatGPT, exemplifying innovative problem-solving abilities in the light of entrepreneurial traits, itself is an example of entrepreneurship and has the ability to influence many stakeholders. At the same time, when this artificial intelligence application is evaluated as an entrepreneur candidate, it has been seen that ChatGPT-4 can effectively address a significant portion of entrepreneurship exam questions. In other words, ChatGPT-4, with its self-learning capabilities, represents a transformative tool in the field of AI and has the potential to be both an entrepreneurial example and an entrepreneurial candidate by gaining more and more talent every day.

This research focused on examining the ChatGPT-4 version, a well-known AI application with self-learning capabilities, within the context of business establishment processes. By combining the Entrepreneurship Handbook questions with the ChatGPT-4 model, the study analysed the model's problem-solving abilities and the originality of its answers compared to the entrepreneurship literature. The research found that ChatGPT-4, as an exemplary entrepreneurship AI model, successfully answered the questions posed in the Entrepreneurship Handbook, demonstrating deep analysis and creativity in developing alternative solutions. This study pioneers the exploration of the relationship between artificial intelligence and entrepreneurship. The research examined the Entrepreneurship Handbook, which consists of 16 chapters covering different aspects of entrepreneurship. Using content analysis, this study evaluated the effectiveness of ChatGPT-4 in addressing entrepreneurship-related questions.

All 160 questions in the handbook were analysed, and ChatGPT-4 demonstrated its ability to provide accurate answers. The data analysis process involved three stages: initial analysis with the dataset, re-analysis of incorrect answers with language exchange, and final analysis of incorrect answers with content assistance. In the first stage, it was able to answer 91.25% of all questions correctly. Upon completion of the second stage, this rate increased to 93.75%. At this point, the most notable issues were ChatGPT-4's comments on the use of language in some questions. For example, ChatGPT-4 stated that there were expressions that could create ambiguity in his answer to a question written in the mother tongue. This showed that the artificial intelligence application has superior language capacity in different languages. Its language usage skills and capacity to correctly understand and solve questions were found to be very important and meaningful. Adapting artificial intelligence applications to everyday devices and equipment holds the potential to enhance various aspects of daily life, such as improving efficiency, convenience, and accessibility. It is essential to recognize the potential ethical implications associated with the deployment of artificial intelligence applications, as evidenced by findings from the data set analysis. Instances such as privacy concerns, bias in decision-making, and misuse of personal data highlight the need for ethical oversight and regulation in AI development and implementation. Overall, this research highlights the potential of AI in addressing entrepreneurship-related questions and generating original solutions. The findings contribute to the understanding of the intersection between artificial intelligence and entrepreneurship, particularly within the context of the literature.

In related literature there are many interdisciplinary studies dealing with the capabilities of ChatGPT. For example, one of the researches claims that professional licensing exam, also known as “the Bar Exam,” is almost universally required in the US as a prerequisite for the practice of law and the famously difficult common core of US professional legal accreditation examinations was determined to be passable by this artificially intelligent tool ( Bommarito II & Katz, 2022). One another study dealing with how definitions of crowdsourcing, alternative finance, and community finance differ from or agree with responses provided by actual people in academic literature ( Wenzlaff & Spaeth, 2022) while a medical paper discusses the contributions of ChatGPT’s coding assistance ( Shen et al., 2023). Based on the findings and methodology outlined in the research, several predictions can be made for future studies on artificial intelligence and entrepreneurship. Future studies could examine and compare different AI models, such as GPT-5 or other advanced models, to assess their effectiveness in addressing entrepreneurship-related questions, comparisons between different models could provide insights into their performance, strengths, and limitations. Future studies could investigate the impact of different languages on AI model performance. Also, examining how language variations affect the accuracy and effectiveness of AI models in entrepreneurship-related tasks can help researchers gain a better understanding of linguistic influences. The research mentioned the self-learning capabilities of ChatGPT-4. Future studies could conduct long-term assessments to understand how AI models continuously improve their performance over time through self-learning. Tracking the model's learning progress and evaluating its ability to adapt and provide more accurate answers can offer valuable insights into the potential of AI for entrepreneurship-related tasks. Besides that, given the success of ChatGPT-4 in addressing questions from the Entrepreneurship Handbook, scholars can explore the integration of AI models in entrepreneurship education. Designing AI-powered tools or platforms that provide real-time feedback, suggestions, and explanations to aspiring entrepreneurs can enhance their learning experiences and problem-solving abilities.

Future studies should prioritize investigating the ethical considerations and potential biases associated with AI models in entrepreneurship. Assessing the fairness and inclusivity of AI systems is essential to mitigate any unintended biases and ensure that AI tools contribute to a diverse and equitable entrepreneurial ecosystem. Interdisciplinary collaboration is strongly recommended for scholars interested in studying artificial intelligence and entrepreneurship. Artificial intelligence and entrepreneurship are multifaceted fields, and collaborating with experts from various disciplines, including computer science, business, and psychology, can offer a more comprehensive understanding of their intersection. Foster interdisciplinary collaboration to leverage diverse perspectives and expertise. By adhering to these recommendations and focusing on the evolving landscape of AI and entrepreneurship, scholars can significantly contribute to the advancement of knowledge and understanding in this dynamic and rapidly evolving field.

## Ethical considerations

Ethical approval and written consent were not required.

## Data Availability

Figshare: Entrepreneurship Questions.docx, https://doi.org/10.6084/m9.figshare.25303393.v1 ( Saygın, 2024). Data are available under the terms of the Creative Commons Attribution 4.0 International license (CC-BY 4.0). figshare: Artificial Intelligence model Chatgpt-4: entrepreneur candidate and entrepreneurship example, Prisma Checklist, https://doi.org/10.6084/m9.figshare.25550961
