# Supplementary material for: Artificial Intelligence Model Chatgpt-4: Entrepreneur Candidate and Entrepreneurship Example
Source: F1000Res. 2024 May 22;13:308. Originally published 2024 Apr 22. [Version 2] doi: 10.12688/f1000research.144671.2 (PMC11153998; doi:10.12688/f1000research.144671.2)
Supplement: Peer review history

# Reviewer report: Reviewer response for version 2

**DOI:** 10.5256/f1000research.166785.r284139

**Published:** 2024-06-10

**Authors:** Tahseen Anwer Arshi, Prof. Venkoba Rao

**Affiliations:** 1 American University of Ras Al Khaimah, Ras Al Khaimah, United Arab Emirates; 2 Majan University College, Ruwi, Muscat Governorate, Oman

The research article makes a unique contribution by exploring the potential of ChatGPT4 in addressing entrepreneurial questions, a significant issue in the field of AI and Entrepreneurship. The authors' attempt to bridge the gap between AI and Entrepreneurship is commendable. The study concludes that ChatGPT4 provides alternatives to the correct answers specified in the entrepreneurship handbook. Overall, the study shows academic merit as it investigates a pertinent issue that needs to be redressed.

**Introduction**

 The introduction comprises three main themes: scientific and technological developments, entrepreneurship development and contributions, and Turkey's entrepreneurship support program (KOSGEB).

 There is a need for multiple improvements in the introduction. The discussion on entrepreneurship is too generic. It attempts to cover various aspects, such as the role of entrepreneurship in the public and private spectrum, its link to innovation, and women in entrepreneurship. It must be more concise by discussing the relevant aspects related to the study.

 Further, the authors should have highlighted the need for this study, potential research gaps, and contributions it makes to theoretical and practical knowledge. While discussing the above suggestions, the authors could demonstrate better integration and synthesis across the three themes. The research gaps and the rationale for the study should lead to precise hypotheses and a more focused discussion on entrepreneurship that requires attention.

**Literature review**

 The literature review covering the emergence, growth, and application of AI in detail is exemplary. However, what is conspicuously missing is the review related to entrepreneurship and the potential need for using AI in answering critical entrepreneurial questions and, more broadly, solving entrepreneurial problems.

 The authors could have discussed various known characteristics of entrepreneurs, such as out-of-the-box thinking and innovative problem-solving, which could have determined why AI is required or if AI can think like an entrepreneur. The authors cite a few relevant works demonstrating their awareness of related literature. The authors can refer to Davidsson and Sufyan (2023) (Ref - 3), Shepherd and Majchrzak (2022) (Ref- 7), Arshi et al. (2021) (Ref -1), Iansiti and Lakhani (2020) (Ref -5), Townsend and Hunt (2019) (Ref- 8), and Kamishima et al. (2018) (Ref- 6) to strengthen the literature review.

 The recency is a suggested area for improvement. A few of the cited works are dated but relevant. Considering the currency of the ChatGPT4, more recent works could be included in the study. For example, it is worth referring to Javaid et al. (2023) (Ref- 4), Badini et al. (2023) (Ref-2), and Wang et al (2023) (Ref-9).

 Generally, the language of the literature review is clear. The jargon is minimal. However, statements such as - "ChatGPT - 4 can successfully answer entrepreneurship exam questions in Turkey without prior knowledge are ambiguous and require correction as they might hinder the reader's understanding. Questions might arise in the reader's mind: What do "Success" and "Prior Knowledge" refer to?

**Methods**

 In the "Methods" section, it is stated that the research approach adopted is content analysis. A proper explanation should have been given for why this was chosen and its suitability for addressing the research questions. There is a lack of clarity in the statement at the end of the paragraph that the census approach is preferred. Moreover, the discussion on study design and data collection starts with data analysis, which must be corrected.

 The authors state four hypotheses in the methods sections. However, they can revisit the language of the hypothesis as they are pretty descriptive and do not show the relationship between independent and dependent variables, especially in hypothesis 2. Further, the second hypothesis states that - ChatGPT-4 can employ the self-learning method in the entrepreneurship exam. Justification on why entrepreneurship exams differ from the normal ones must be included. Finally, there is an overlap between hypotheses 1 and 4.

**Findings**

 The authors should exercise caution while generalizing the results of this study. It is stated that ChatGPT is an outstanding entrepreneur. Such a conclusion cannot be reached if it just answers a set of questions. It is essential that the authors accurately interpret the statistical findings. The authors do not refer to the hypotheses in the findings, which should have been done.

**Discussion and Conclusions**

 The current title of the section in the paper is "Conclusion and Discussion," which should be the other way around. While this study fills a gap in the existing literature, there is a need for further research. A section on the study's practical implications should be added before the conclusion. A brief paragraph can be added on the future areas that researchers can investigate based on the gaps that stem from this research.

**References: **(Ref 1-9)

- **Is the work clearly and accurately presented, and does it cite the current literature?**

Partly, the study is clearly presented but there is room for improvement in integration and synthesis especially the Introduction section. Moreover, it could benefit from the recent literature that has been suggested by the reviewers.

- **Is the study design appropriate, and does the work have academic merit?**

Yes, the study design is appropriate and thorough and could improve with the justification of the chosen methods.

- **Are sufficient details of methods and analysis provided to allow replication by others?**

Yes, the authors provide sufficient details of methods and analysis.

- If applicable, is the statistical analysis and its interpretation appropriate?

Yes, the statistical analysis and its interpretation are appropriate.

- **Are all the source data underlying the results available to ensure full reproducibility?**

Partly, since measures are standard and results are outputs from a computer program, they can be reproduced with some variability. Additionally, the entrepreneurial dimensional are mainly based on the Turkish context.

- **Are the conclusions drawn adequately supported by the results?**

Yes, the conclusions drawn are adequately supported by the results.

Is the work clearly and accurately presented and does it cite the current literature?

Partly

If applicable, is the statistical analysis and its interpretation appropriate?

Yes

Are all the source data underlying the results available to ensure full reproducibility?

Partly

Is the study design appropriate and is the work technically sound?

Yes

Are the conclusions drawn adequately supported by the results?

Yes

Are sufficient details of methods and analysis provided to allow replication by others?

Yes

Reviewer Expertise:

Entrepreneurship, Machine Learning, and AI

We confirm that we have read this submission and believe that we have an appropriate level of expertise to confirm that it is of an acceptable scientific standard, however we have significant reservations, as outlined above.

## References

[1] : SECURE – a new business model framework for measuring start-up performance. *Journal of Entrepreneurship in Emerging Economies* .2021;13(3) : 10.1108/JEEE-02-2020-0043 459-485 10.1108/JEEE-02-2020-0043

[2] : Assessing the capabilities of ChatGPT to improve additive manufacturing troubleshooting. *Advanced Industrial and Engineering Polymer Research* .2023;6(3) : 10.1016/j.aiepr.2023.03.003 278-287 10.1016/j.aiepr.2023.03.003

[3] : What does AI think of AI as an external enabler (EE) of entrepreneurship? An assessment through and of the EE framework. *Journal of Business Venturing Insights* .2023;20: 10.1016/j.jbvi.2023.e00413 10.1016/j.jbvi.2023.e00413

[4] : A study on ChatGPT for Industry 4.0: Background, potentials, challenges, and eventualities. *Journal of Economy and Technology* .2023;1: 10.1016/j.ject.2023.08.001 127-143 10.1016/j.ject.2023.08.001

[5] : Competing in the Age of AI: Strategy and Leadership when Algorithms and Networks Run the World. *Harvard Business Review Press* .2020;

[6] : Can Merging a Capability Approach with Effectual Processes Help Us Define a Permissible Action Range for AI Robotics Entrepreneurship?. *Philosophy of Management* .2018;17(1) : 10.1007/s40926-017-0059-9 97-113 10.1007/s40926-017-0059-9

[7] : Machines augmenting entrepreneurs: Opportunities (and threats) at the Nexus of artificial intelligence and entrepreneurship. *Journal of Business Venturing* .2022;37(4) : 10.1016/j.jbusvent.2022.106227 10.1016/j.jbusvent.2022.106227

[8] : Entrepreneurial action, creativity, & judgment in the age of artificial intelligence. *Journal of Business Venturing Insights* .2019;11: 10.1016/j.jbvi.2019.e00126 10.1016/j.jbvi.2019.e00126

[9] : Chat with ChatGPT on Industry 5.0: Learning and Decision-Making for Intelligent Industries. *IEEE/CAA Journal of Automatica Sinica* .2023;10(4) : 10.1109/JAS.2023.123552 831-834 10.1109/JAS.2023.123552

# Reviewer report: Reviewer response for version 2

**DOI:** 10.5256/f1000research.166785.r281452

**Published:** 2024-06-05

**Authors:** Ivan Diryana Sudirman

**Affiliations:** 1 BINUS Business School Undergraduate Program, Bina Nusantara University, Bandung, Indonesia

I have no further comments

Is the work clearly and accurately presented and does it cite the current literature?

Partly

If applicable, is the statistical analysis and its interpretation appropriate?

No

Are all the source data underlying the results available to ensure full reproducibility?

Yes

Is the study design appropriate and is the work technically sound?

Yes

Are the conclusions drawn adequately supported by the results?

Yes

Are sufficient details of methods and analysis provided to allow replication by others?

Yes

Reviewer Expertise:

Management Information system, data science, marketing

I confirm that I have read this submission and believe that I have an appropriate level of expertise to confirm that it is of an acceptable scientific standard.

# Reviewer report: Reviewer response for version 2

**DOI:** 10.5256/f1000research.166785.r286722

**Published:** 2024-06-05

**Authors:** Muhammad Nadeem

**Affiliations:** 1 University of Education, Lahore, Pakistan

This article provides a comprehensive analysis of the capabilities and implications of artificial intelligence (AI), particularly focusing on the ChatGPT-4 model within the context of entrepreneurship .

 The methodology section outlines a 3-stage process employed

 to ascertain the accuracy of solutions to the questions. These stages primarily involve altering the query expressions directed towards ChatGPT. Initially, in the first stage as specified in the method section, the questions were presented to ChatGPT with the designated query expression, and responses were solicited.

 The initial findings revealed that 144 out of 160 questions received correct answers. Subsequently, the 16 questions with incorrect responses proceeded to the second stage, which involved reanalyzing

 each question, considering alternative answers, and providing the most appropriate response along with its rationale.

 Following this stage and the adjustment of the query, it was observed that an additional 6 questions were answered correctly. In the third

 stage, a new query expression was introduced, prompting ChatGPT to "Carefully read the paragraph. Rethink like an aspiring entrepreneur and a scholar specializing in entrepreneurship. Analyze each question below thoroughly and provide the most appropriate

 answer along with its rationale.” This approach resulted in the successful resolution of all remaining incorrectly answered questions, as determined by the findings.

Is the work clearly and accurately presented and does it cite the current literature?

Yes

If applicable, is the statistical analysis and its interpretation appropriate?

Yes

Are all the source data underlying the results available to ensure full reproducibility?

Partly

Is the study design appropriate and is the work technically sound?

Yes

Are the conclusions drawn adequately supported by the results?

Yes

Are sufficient details of methods and analysis provided to allow replication by others?

Partly

Reviewer Expertise:

Quantitative analysis, Social sector

I confirm that I have read this submission and believe that I have an appropriate level of expertise to confirm that it is of an acceptable scientific standard.

# Reviewer report: Reviewer response for version 1

**DOI:** 10.5256/f1000research.158505.r275011

**Published:** 2024-05-15

**Authors:** Ivan Diryana Sudirman

**Affiliations:** 1 BINUS Business School Undergraduate Program, Bina Nusantara University, Bandung, Indonesia

The application of ChatGPT in the setting of entrepreneurial processes is investigated in this paper. ChatGPT is integrated into the study to address questions across 16 modules using the Entrepreneurship Handbook published by Turkey's SME Development Organization. This approach enables a comparison of solutions produced by AI with well-established entrepreneurship literature. The results show how well ChatGPT does in offering fresh, in-depth examinations of the questions in the handbook and how imaginatively it proposes fresh solutions to the given ones. Because it pioneers the nexus of artificial intelligence and entrepreneurship research, the study is important.

 Some references use the year 2018 and below, such as Russell & Norvig, 2010.

 This research is technically sound.

 Method is clear and simple, allowing for replication easily.

 No need for statistical analysis because it's just like giving an exam on ChatGPT and then seeing the test results.

 While the conclusion of the research is well articulated, it is embedded within the discussion section rather than being presented in a distinct conclusion section. Typically, conclusions should be presented in a dedicated section to clearly summarize the findings and implications.

Is the work clearly and accurately presented and does it cite the current literature?

Partly

If applicable, is the statistical analysis and its interpretation appropriate?

No

Are all the source data underlying the results available to ensure full reproducibility?

Yes

Is the study design appropriate and is the work technically sound?

Yes

Are the conclusions drawn adequately supported by the results?

Yes

Are sufficient details of methods and analysis provided to allow replication by others?

Yes

Reviewer Expertise:

Management Information system, data science, marketing

I confirm that I have read this submission and believe that I have an appropriate level of expertise to confirm that it is of an acceptable scientific standard, however I have significant reservations, as outlined above.
